# Editorial: Metabolomics: a tool to understand plant protection against biotic and abiotic stresses

**DOI:** 10.3389/fpls.2023.1274405

**Published:** 2023-08-24

**Authors:** Laura Cuyas, Adrián Schwarzenberg

**Affiliations:** ^1^ Staphyt, Inchy-en-Artois, France; ^2^ Analytical Chemistry Core Lab (ACL), King Abdullah University of Science and Technology, Thuwal, Saudi Arabia

**Keywords:** plant protection, metabolomics, agriculture, biotic stress, abiotic stress

The world population is projected to grow by 30%, reaching 9.7 billion by 2050. To ensure global food security, the Food and Agriculture Organization of the United Nations (FAO) estimates that world food production should increase by 70% (Molotoks et al., 2018). However, achieving these objective faces numerous challenges, including plant pests and abiotic stresses such as global warming, droughts, torrential rains, soil salinity, and light intensity. These factors make it difficult to meet the increased demand.

Since the Green Revolution in the 1960s, chemicals like fertilizers and pesticides have been widely used to enhance agricultural yields. However, their negative impact on the environment has been well-documented (Jacquet et al., 2022; Mukherjee, 2022). As a result of growing concerns about sustainability and environmental protection, the European Commission has set an ambitious target under the EU Green Deal: reducing overall chemical use and hazardous pesticide risk by 50% before 2030.

To overcome these challenges while minimizing harm to nature, it is essential to develop new strategies that incorporate both conventional products (primarily chemicals) and non-conventional alternatives such as biologicals [biopesticides (Hernandez-Tenorio et al., 2022) and biostimulants (Gonzalez-Perez et al., 2021; Ma et al., 2022)], along with innovative technologies (Gonzalez Guzman et al., 2022).

To achieve this goal effectively, researchers are turning towards omics approaches including genomics, transcriptomics, proteomics, and metabolomics (Hall, 2011). By identifying altered metabolic pathways caused by various biotic and abiotic stresses alongside characterizing newly developed products’ modes of action through comprehensive studies—significant progress can be made in plant protection (Nakabayashi and Saito, 2015).

Within this Research Topic focused on metabolomics approaches for understanding plant protection mechanisms:

Rodrígues Neto et al., conducted one of the first multi-omic integration analyses on Purslane plants (*Portulaca oleracea L*.) subjected to salinity stress mitigation—a critical factor considering Purslane’s adaptability worldwide due to its medicinal properties. Another study performed by Dai et al., explored cross-pollination effects on fruit set and weight in pitaya (*Hylocereus polyrhizus*). Researchers discovered that low-temperature storage negatively affects pollen quality and germination rate, shedding light on the importance of investigating long-term pollen storage techniques.

Wu et al. delved into the impact of shading on blueberries to counteract extreme heat stress during their peak production period. Moderate shading (50%) was found to enhance plant growth, enrich photosynthetic pathways, and promote flavonoid biosynthesis—an effective strategy for improving blueberry cultivation under hot temperatures.

Addressing herbicide resistance in weeds, Yang et al. identified key genes responsible for P450-mediated metabolic-herbicide resistance against Asia minor bluegrass (*Polypogon fugax*) commonly found in China’s wheat, rice, or maize fields—a pressing issue with substantial implications for crop management.

Lastly, Tran et al., through untargeted metabolomics analyses on *Arabidopsis thaliana* plants treated with seaweed extracts from *Durvillaea potatorum* and *Ascophyllum nodosum*, characterized the metabolic changes promoting plant growth and activating defense systems locally or systemically providing valuable insights for developing new biostimulant and biocontrol products derived from algae.

These findings contribute significantly to advancing our understanding of specific metabolites and metabolic pathways involved in plant protection while offering potential solutions for sustainable agriculture moving forward.

In summary, addressing the challenges of global population growth and food security requires sustainable agricultural strategies. The use of chemicals in farming has raised environmental concerns, prompting researchers to explore innovative approaches like plant metabolomic studies. The Research Topic highlighted insights into adaptive mechanisms under salinity stress in non-conventional plants, pollen storage for cross-pollination success, shading techniques for enhanced blueberry growth in extreme heat, herbicide resistance mechanisms, and the characterization of metabolic changes induced by seaweed extracts. By integrating these findings into agricultural practices and leveraging technology, we can achieve higher yields while reducing reliance on harmful chemicals—creating a resilient and sustainable future for global food security without compromising our environment.

## Author contributions

AS: Conceptualization, Writing – review & editing. LC: Conceptualization, Writing – review & editing.
